# Intricate Connections between the Microbiota and Endometriosis

**DOI:** 10.3390/ijms22115644

**Published:** 2021-05-26

**Authors:** Irene Jiang, Paul J. Yong, Catherine Allaire, Mohamed A. Bedaiwy

**Affiliations:** Division of Reproductive Endocrinology and Infertility, Department of Obstetrics and Gynecology, University of British Columbia, D415A-4500 Oak Street, Vancouver, BC V6H 3N1, Canada; irene.jiang@cw.bc.ca (I.J.); paul.yong@vch.ca (P.J.Y.); callaire2@cw.bc.ca (C.A.)

**Keywords:** endometriosis, microbiota, dysbiosis, estrogen, estrobolome, metabolome, Lactobacillus, vaginal microbiota, uterine microbiota, gut microbiota, inflammation, immune dysregulation, antibiotics, probiotics

## Abstract

Imbalances in gut and reproductive tract microbiota composition, known as dysbiosis, disrupt normal immune function, leading to the elevation of proinflammatory cytokines, compromised immunosurveillance and altered immune cell profiles, all of which may contribute to the pathogenesis of endometriosis. Over time, this immune dysregulation can progress into a chronic state of inflammation, creating an environment conducive to increased adhesion and angiogenesis, which may drive the vicious cycle of endometriosis onset and progression. Recent studies have demonstrated both the ability of endometriosis to induce microbiota changes, and the ability of antibiotics to treat endometriosis. Endometriotic microbiotas have been consistently associated with diminished *Lactobacillus* dominance, as well as the elevated abundance of bacterial vaginosis-related bacteria and other opportunistic pathogens. Possible explanations for the implications of dysbiosis in endometriosis include the Bacterial Contamination Theory and immune activation, cytokine-impaired gut function, altered estrogen metabolism and signaling, and aberrant progenitor and stem-cell homeostasis. Although preliminary, antibiotic and probiotic treatments have demonstrated efficacy in treating endometriosis, and female reproductive tract (FRT) microbiota sampling has successfully predicted disease risk and stage. Future research should aim to characterize the “core” upper FRT microbiota and elucidate mechanisms behind the relationship between the microbiota and endometriosis.


**Term**

**Definition**
MicrobiotaThe collection of all the microorganisms residing in and on the body, including bacteria, archaea, protists, fungi, and virusesMicrobiomeThe aggregate of all the genetic material of the microbiotaEstrobolomeThe total collection of genes, in the gut microbiota, responsible for estrogen metabolismMetabolomeThe total collection of metabolites in a given environmentDysbiosisImbalance or impairment of the microbiota, characterized by gain of pathogenic microbes or loss of probioticsPrebioticCompounds that promote growth and activity of beneficial microorganismsProbioticLive microorganisms that are beneficial to host health

## 1. Endometriosis

### 1.1. Introduction of Endometriosis

Endometriosis is a complex gynaecological disease characterised by the presence of endometrial glands and stroma outside the uterus [1]. This tissue is often found at sites in the pelvis, including ovaries, fallopian tubes, peritoneal surfaces, the bowel and bladder, but can also engraft in distant organs [1,2]. Much like the eutopic endometrium, these histologic lesions respond to estrogen and are driven to proliferate and bleed alongside the menstrual cycle [1]. Thus, the disease primarily manifests between menarche and menopause, affecting approximately 10% of reproductive-aged women [1,3,4,5]. However, the true prevalence of endometriosis remains enigmatic because the condition presents differently across patients, ranging from symptomatic to asymptomatic independent of its severity, and reliable non-invasive tests are not yet available [2,6]. 

Like the uterine lining, endometriotic implants bleed during menstruation, activating local inflammation and inducing pelvic pain [1,2]. Often chronic, the disease can have significant impact on a woman’s physical, mental, sexual and social wellbeing [7,8,9,10]. Prolonged inflammation at the lesions can lead to formation of adhesions and scarring (fibrosis), as well as debilitating symptoms including severe pelvic pain, dysmenorrhea, dyspareunia, dyschezia and subfertility [1,11]. Symptoms can be relieved by surgically excising the peritoneal implants, or by supressing lesion growth and bleeding through hormonal modulation [12,13]. Although many management approaches enhance fertility and relieve pain, the benefit is moderate and associated with high recurrence rates and side-effects of hormonal therapy and risks of surgery [1,14]. The current standard of clinical diagnosis involves surgical visualisation [2], making it not only costly and invasive to diagnose, but also limits our ability to study it in the asymptomatic general population. 

### 1.2. Aetiology and Pathogenesis

**Figure 1:**
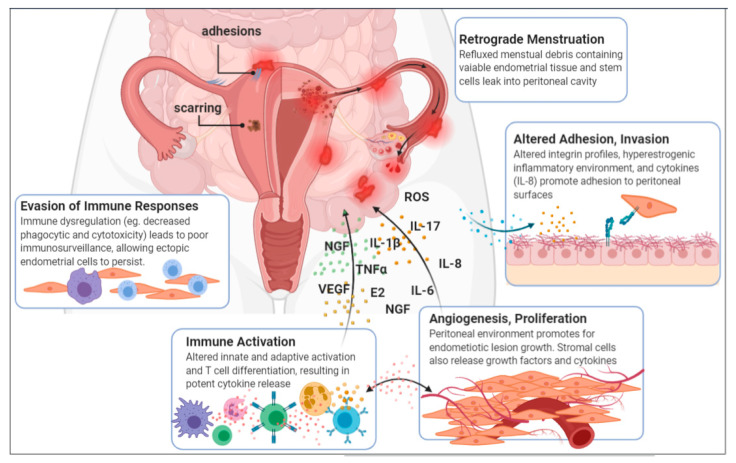
Aetiology and pathogenesis of endometriosis.

Endometriosis is a multifactorial disease, and its aetiology and pathogenesis are still ill-established. One of the most widely accepted theories on the origin of ectopic endometrial tissues is “Retrograde Menstruation”, which refers to the reflux of menstrual debris with viable endometrial cells via the fallopian tubes into the pelvic cavity [1,15,16]. Once there, cells in the endometrial deposits must adhere to peritoneal surfaces and proliferate in order to develop into invasive lesions (Figure 1) [17,18]. Endometrial stromal cells from women with endometriosis display increased adhesive properties as a result of altered integrin profiles, allowing them to adhere to the peritoneal lining [18,19]. Cellular adhesion is further enhanced by the inflammatory peritoneal environment, which is a hallmark of endometriosis. For example, abundantly present pro-inflammatory cytokine interleukin-8 (IL-8) stimulates cells to adhere to extracellular proteins [20], thus regulating the initial establishment of the disease. 

To survive and expand, endometriotic implants require a blood supply. The process of angiogenesis is regulated by various angiogenic factors, such as vascular endothelial growth factor (VEGF), which has upregulated expression in the peritoneal fluid of patients with endometriosis [18,21,22]. VEGF in the peritoneal fluid (PF) is primarily produced by macrophages, and its expression is directly regulated by estradiol and progesterone [23]. Tumor necrosis factor-α (TNF-α) and IL-8, also secreted by peritoneal macrophages, are other potent inducers of angiogenesis and lesion proliferation [24,25]. TNF-α is a predominant product of activated macrophages, which stimulates other leukocytes to produce IL-6 and more TNF-α. Its role in stimulating endometrial cell adhesion and inducing angiogenesis is necessary in the initial stages of endometriosis establishment [26]. In addition, excessive pelvic blood leads to the generation of reactive oxygen species (ROS), which cause tissue damage and exposes tissues, favoring angiogenesis [11].

The persistence of endometrial debris in the peritoneum may overload the immune system, causing low-grade inflammation, and over time possibly lead to chronic immune dysregulation [27]. This results in poor immunosurveillance, allowing the foreign tissue to escape immune defenses, and has immense consequences for endometriosis [21], as we review below. 

### 1.3. A Disease of the Immune System

In endometriosis, the peritoneal environment is in a chronic state of local inflammation, and contains immune cells with altered functions. This immune dysregulation in endometriosis creates an ideal environment for disease progression [21]. At present, it is unclear whether immune dysfunction is a pathophysiological *hallmark* or *cause* of endometriosis. In either case, there is a strong association demonstrated by the following findings. Table 1 summarizes how immune dysregulation is embodied in the major types of immune cells involved. 

#### 1.3.1. Elevated Inflammatory Mediators

Cytokines and prostaglandins are key players in the initiation, propagation and regulation of immune responses, including inflammation processes. Surges of cytokines lead to signaling cascades and activation of immune cell activity, recruiting more immune cells, and leading to further cytokine production. In the peritoneum, these molecules are produced by various leukocytes, mainly macrophages and stromal cells of the ectopic endometrial tissues, which elicit a localised immune and inflammatory response [2,21,28]. The stromal cells produce IL-6 at similar rates as macrophages, and have further increased production when stimulated by TNF-α [29]. It was found that in women with endometriosis, even eutopic endometrial cells produce higher quantities of IL-6 under basal conditions when compared to women with endometriosis [30]. Furthermore, events that occur in women with endometriosis such as overexpression of NF-κB by peritoneal macrophages and endometriotic cells, activation of MAPK pathways and production of ROS all contribute to cytokine production [31]. Additionally, women with endometriosis have been reported to have increased numbers of immune cells in the PF, which secrete various growth factors and cytokines, enhancing survival and proliferation of the ectopic endometrial cells [18,32]. Another study investigating plasma inflammatory markers found that elevated plasma levels of IL-1β and TNF-α were associated with increased risk for endometriosis [32]. As a result of all this, the PF of women with endometriosis is a potent mixture of cytokines whose positive feedback mechanism maintains a chronic state of inflammation. 

#### 1.3.2. Macrophages: Principal Contributors to the Pathogenesis of Endometriosis

The peritoneal fluid of women with endometriosis contains a higher number of activated macrophages in comparison to healthy controls, and these immune cells are postulated to be the primary contributors to the pathogenesis of endometriosis, in part due to their high level of cytokine secretion [18,33]. They are recruited to the peritoneal cavity by various chemo-attractants, including IL-8, and are the main source of IL-6 [18]. Their activity produces the perfect environment for the adhesion, survival and progression of ectopic endometrial implants [18,33,34,35]. 

These important immune cells phagocytose pathogens, present antigen, and play a critical role in tissue regeneration, angiogenesis and wound healing [36]. In the healthy endometrium, their numbers fluctuate throughout the menstrual cycle, heightening in the secretory phase [37]. This allows them to phagocytose cell debris and apoptotic cells, effectively cleaning up after endometrial shedding. However, this normal fluctuation is not observed in women with endometriosis, which could contribute to the survival ability of refluxed endometrial cells in the peritoneum [33,38]. 

Furthermore, the peritoneal macrophage population in endometriotic women are phenotypically distinct; they exhibit *decreased* phagocytic capacity and *increased* activation of NF-κB pathways, leading to the downstream upregulation of proinflammatory cytokines (TNF-α, IL-1β, and IL-6), proangiogenic factors (VEGF), growth factors and adhesion molecules [2,21,33,39,40]. Macrophages can be phenotypically categorised as “classically activated” (M1) or “alternatively activated” (M2), and their polarisation state depends on their microenvironment [33]. M1 are involved in proinflammatory responses, while M2 are involved in anti-inflammatory responses, tissue repair and angiogenesis [38]. A recent study revealed that in women with endometriosis, peritoneal M1 exhibited exaggerated proinflammatory qualities and M2 tended to switch toward the proinflammatory phenotype of M1 [33]. This supports important previous findings in mice that macrophages infiltrating endometriotic lesions express markers of activation and are necessary for lesion growth and vascularisation [35]. However, the mechanisms of macrophage plasticity are still under debate. Nonetheless, these findings suggest that peritoneal macrophages of women with endometriosis have reduced ability to clear out invasive endometriotic cells, and instead contribute to their growth. 

#### 1.3.3. Preconditioned Neutrophils

The PF of women with endometriosis also contains higher numbers of neutrophils, recruited by potent chemoattractant IL-8 and preconditioned by bacterial presence [21,41,42]. A study found that neutrophil infiltration in ectopic endometrial tissues peaked in the early stages of lesion formation and subsequently declined, indicating an important role for neutrophils in early lesion formation [34]. 

#### 1.3.4. Impaired Natural Killer Cells

The peritoneal immune environment in endometriosis patients is known to impair natural killer (NK) cell activity, and is an example of immune dysregulation in endometriosis [21]. NK cells in diseased women express altered patterns of activating and inhibitory receptors, and display reduced cytotoxicity when exposed to IL-6 and transforming growth factor beta (TGF-β) [21,43]. This immunosuppressive activity partially explains how ectopic endometrial cells are able to evade immunosurveillance and persist in the peritoneal cavity [21]. 

#### 1.3.5. Altered T cell Differentiation

T cell subset profiles are altered in women with endometriosis [21]. Cytokine secretion by T helper (T_H_) cells is shifted toward T_H_2, which is involved in the suppression of cell-mediated immunity, potentially lending to poor immunosurveillance [44,45]. There are also higher numbers of T_H_17 cells in the PF of endometriosis patients, and consequently higher concentrations of IL-17 [46]. The presence of elevated T_H_17 cells and IL-17 plays an established role in promoting chronic inflammation [32,47]. IL-17 stimulates production of cytokines that induce angiogenesis and inflammation, contributing to the progression of endometriosis [48].

#### 1.3.6. Activated B Cells

B cells are also implicated in endometriosis, although their role is mostly speculative [21]. They are known to produce anti-endometrial autoantibodies, IL-6 and IL-17, which contribute to inflammation [21,49,50]. 

It is evident that peritoneal immune dysfunction is deeply involved in endometriosis, and accumulating evidence suggests that presence of pathogenic, non-commensal bacteria in the gut and uterine microbiome may be a contributing factor. 

### 1.4. Estrogen Levels and Signaling Is Altered in Endometriosis 

Estrogen is heavily involved in many aspects of endometriosis, and the disease is also considered a hormone-dependent disease, as it bears symptoms restricted to the reproductive period and is responsive to hormonal treatment [47,51]. In fact, a 2017 study found that estrogen is necessary to induce endometriosis [52]. In women, estrogen stimulates the growth of ectopic endometrial tissues and inflammatory activity, and endometriosis has been associated with alterations in estrogen signaling [47]. For instance, endometriotic women have a heightened proinflammatory and anti-apoptotic response to estradiol [53]. This may be attributed to the changes in nuclear estrogen receptor expression. 

Endometriotic lesions express higher levels of estrogen receptor-β (ER-β), whose signaling promotes lesion growth by inhibiting TNF-α-induced apoptosis, activating an inflammasome which increases IL-1β, and enhancing cellular adhesion and proliferation [54]. In this study, they found that TNF-α, detected abundantly in the peritoneum of women with endometriosis, cooperates indirectly with ER-β to incite these events [54]. In murine models, expression of nuclear estrogen receptors (ER-α and ER-β) is altered in lesions, and this ER signaling is necessary for lesion establishment [55]. They found that ER-α signaling drove proliferation, adhesion and angiogenesis of ectopic lesions [55]. 

Another consequence of estrogen in endometriosis is its ability to affect peripheral nerve fibres directly or indirectly through the upregulation of various growth factors, including nerve growth factors (NGF), contributing to nociceptive pain [56].

Three key factors dysregulating estrogen availability in endometriotic women include expression of estrogen-synthesis enzymes, the estrobolome and the metabolome.

**Figure 2:**
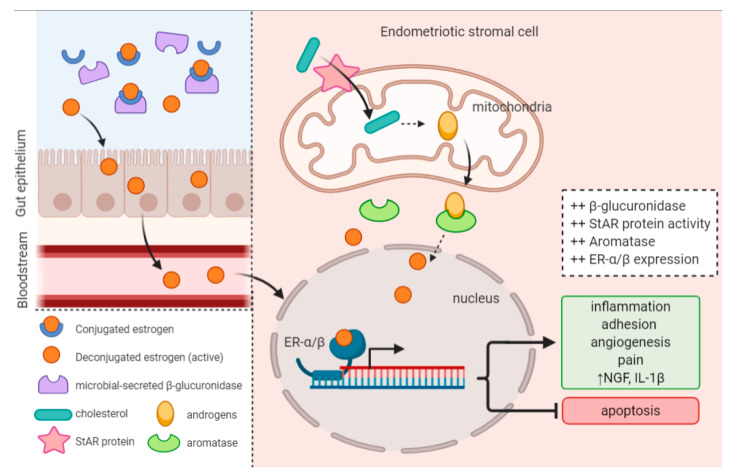
Altered estrobolome activity and upregulated enzyme expression produces a hyperestrogenic environment that promotes endometriosis onset and progression.

In endometriosis, estradiol is made available through systemic hormones and locally in the peritoneal environment through aromatase and steroidogenic acute regulatory protein (StAR) activity [57]. Aromatase is an enzyme that converts androgens into estrogens, and StAR is a transport protein that regulates the transfer of cholesterol in the mitochondria required for steroidogenesis. The upregulated expression of these in endometriotic lesions contributes to the increased availability of estrogen, and drives the disease (Figure 2) [47,57]. In contrast, normal endometrial tissue lacks these enzymes, and is unable to synthesize estrogen [51]. 

Furthermore, estrogen metabolism is known to be regulated by the estrobolome, a collection of genes in the gut microbiome involved in estrogen metabolism [58,59]. Estrobolome activity modulates the amount of excess estrogen that is expelled from or reabsorbed into the body [59]. When this activity is impaired, typically as a result of imbalances in the gut microbiome, excess estrogen can be retained in the body and travel from the gut to the endometrial and peritoneal environment via circulation [59,60]. This contributes to the hyperestrogenic state that drives endometriosis, and provides a possible mechanism as to how dysbiosis in the gut microbiota may be involved in the disease. 

Finally, the metabolome also plays a role in regulating circulating estrogen [61]. The metabolome refers to the total metabolites in a given environment, in this case the gut. It is heavily influenced by gut microbial activity, and includes consequential neuro-active metabolites that affect the brain and its signalling [62,63,64]. This bidirectional link is called the gut-brain-axis, and these compounds bind to host gonadotropin-releasing hormone (GnRH) receptors to stimulate production of luteinising hormone (LH) and follicle-stimulating hormone (FSH), which subsequently stimulate the production of estrogen [61,65].

## 2. The Microbiota

### 2.1. Introduction to the Microbiota 

It is well known that the human microbiota, comprising all the microorganisms living in and on the body, has an immense impact on our wellbeing. From metabolic to immune functions, these diverse microbial communities are vital to human health and alterations or imbalances of the microbiome are a significant cause of disease [66,67]. The mammalian immune system has evolved intricate mechanisms of maintaining homeostasis with resident microorganisms to avoid barrier breech and ensure the host-microbial relationship remains mutualistic [68]. 

### 2.2. Dysbiosis

Dysbiosis is defined as an imbalance or impairment of the microbiota, which can be a combination of increased pathogenic microbes or loss of probiotics, and has remarkable consequences on human health. It has been strongly associated with many diseases such as Inflammatory Bowel Disease, psoriasis, arthritis and cancer [66,67]. Endometriosis shares many similarities with such diseases, and we will soon see how it is impacted by dysbiosis-altered immunoregulatory functions of the microbiota. 

### 2.3. Gut Microbiota

The gut flora is arguably one of the richest and most studied microbiomes, and is known to play an indispensable role in the absorption and synthesis of nutrients, maintenance of mucosal integrity, protection against pathogens and maturation of the immune system [68]. Besides its necessity in maintaining physiologic gastrointestinal function, it has also been found to be a key regulator in many inflammatory and proliferative conditions [69,70,71]. Furthermore, it has been found to affect estrogen metabolism and stem-cell homeostasis [17,59]. In the following sections, we will see how aberrant gut microbiota balance disrupts these important functions and impacts endometriosis. 

#### Role of the Gut Microbiota in Host Immune Function

The gastrointestinal tract is densely populated with organised lymphoid structures housing immune-related cells [68]. It is well known that the gut microbiota plays a major role in the development of these structures and in the development of immune cell function. In fact, in the absence of a gut microbiota, mouse models have shown that such structures do not even develop, and secretory Immunoglobin A (IgA) and cytotoxic T cells are deficient [68]. The gut microbiota also shapes mucosal T cell composition (T_H_1, T_H_17, T_Reg_, etc.), and dysbiosis can upset this delicate balance, triggering inflammation and other diseases [68].

Furthermore, commensal microbes compete for resources, which limits the colonisation of pathogenic microbes. For example, the presence of Lactobacilli in the female reproductive tract prevents *Neisseria gonorrhoeae* from adhering, thus protecting the host from infection [72]. The commensal bacteria also continuously stimulate receptors, leading to upregulation of Toll-like receptors (TLRs), and consequently increased immunosurveillance [73]. 

Bacteria also contribute to healthy barrier development in the gut. For example, *Bacteroides* play a role in tissue regeneration and vascularisation [68]. In addition, bacteria are important for physiologic function of other mucosal surfaces, such as endometrial remodelling in the uterus [73]. During the peri-implantation period, the endometrial epithelium exhibits increased permeability, allowing for breaching of uterine microbiota, and in turn leading to a more pro-inflammatory environment [73]. Secreted metabolites of residential microbes also affect local microenvironments, altering pH or introducing ROS for example [74]. Consequently, these intricate interactions can have clinical consequences if defective. 

### 2.4. Female Reproductive Tract Microbiota

A far less-characterised microbiota exists in the uterine cavity. The upper female reproductive tract (FRT), consisting of the uterus, fallopian tubes and ovaries, was once considered a sterile environment. Although this perception has fundamentally changed over the years, there is still no current consensus on the core female reproductive tract microbiota that exists in healthy women, nor its exact role in endometriosis [39]. However, strong evidence continues to grow in support of this changing perception [75].

#### 2.4.1. Proof of Existence

It is well-established that there is a rich microbiota in the vagina, however, far less is known about the upper FRT microbiota. Up until quite recently, its existence was of debate. Many researchers have isolated bacteria from the endometrium, during hysteroscopy or in murine models, confirming that the uterine environment is certainly not sterile [76]. In fact, up to 95% of hysterectomy samples contain bacterial DNA [77]. In a 2016 study, researchers sought to determine whether microbes in the upper FRT represented a distinct community to the one in the vagina [76]. They identified the existence of a distinct and stable endometrial microbiota by examining bacterial samples from endometrial fluid and vaginal aspirates of reproductive-aged women [76]. 

#### 2.4.2. Female Reproductive Tract Microbiota Composition

Moreno et al. found that the uterine microbial composition differed from the vaginal one, and contained up to 191 operational taxonomic units [76]. When analysed, these microbiotas were categorised as either *Lactobacillus*-dominant (LD, >90% *Lactobacillus* spp.) or non-*Lactobacillus*-dominant (NLD, <90% *Lactobacillus* spp. with >10% of other bacteria) [76]. Moreover, they found that NLD microbiotas were associated with adverse reproductive outcomes. For context, a healthy vaginal microbiota is defined by *Lactobacillus* presence, and imbalances lead to pathologies such has bacterial vaginosis [78]. It is hypothesised that the dominating presence of *Lactobacillus* spp. in the vaginal microbiota lowers the local pH through the production of lactic acid and short-chain fatty acids, prohibiting the growth of pathogenic bacteria [79,80,81]. However, Moreno et al. did not observe a significant association between pH and *Lactobacillus*-dominance [76], suggesting hypothesised mechanisms may be unique to the vaginal environment. They suggest instead that NLD compositions may trigger an inflammatory response in the endometrium, possibly explaining its association with negative pregnancy outcomes [76]. Endometrial inflammation is a hallmark of endometriosis and a major factor in its establishment and progression. It is therefore reasonable to suspect that altered endometrial microbiota (or non-*Lactobacillus*-dominance in this case) may be related. 

Of interest, depletion of *Lactobacillus* and overgrowth of (opportunistic) pathogenic bacteria in the reproductive tract microbiota is characteristic of bacterial vaginosis (BV), a common vaginal inflammatory condition [82,83,84,85,86]. BV increases local levels of pro-inflammatory cytokines and damages the epithelial and mucosal barrier [84,85,87,88,89]. Bacteria associated with BV, such as *Gardnerella*, *Prevotella* and *Bacteroides*, therefore contribute to increased risk of more severe gynaecologic diseases, including endometriosis, PID, endometritis and infertility [74,83,84,90,91,92].

Several researchers have attempted the challenging feat of characterising the uterine microbiota in healthy women compared to those with underlying diseases such as endometriosis. Based on reports to date, the uterine microbiota of healthy, asymptomatic women is most abundantly populated by Firmicutes, Bacteroidetes, Proteobacteria and Actinobacteria [74]. Moreno et al. identified the five most represented genera in reproductive-aged women as *Lactobacillus* (71.7% of identified bacteria), *Gardnerella* (12.6%), *Bifidobacterium* (3.7%), *Streptococcus* (3.2%) and *Prevotella* (0.866%) [76]. Another study identified the existence of *Lactobacillus* spp., *Mycoplasma hominis*, *Gardnerella vaginalis* and *Enterobacter* spp. in the endometrial microbiota [93]. 

## 3. Evidence of an Intricate Connection

Recently, research on the involvement of the microbiota in endometriosis has begun to accrue. It is postulated that dysbiosis may be involved in dysregulating the immune system and altering estrogen metabolism. Having discussed the extensive role of the immune system and estrogen signaling in endometriosis, it would seem inevitable that the microbiota plays a critical role in the disease.

Studies have shown that patients with pelvic inflammatory disease (PID), which results from the ascension of vaginal bacteria up into the uterus, fallopian tubes and ovaries, are associated with a threefold increase in risk of developing endometriosis [94], possibly suggesting that the disease may have an infectious etiology, at least in part [39].

Researchers have found evidence suggesting the gut and female reproductive tract microbiota may be inextricably linked to the onset and progression of endometriosis. This novel perspective on endometriosis opens the door to many preventative, diagnostic and therapeutic possibilities, and is an emerging area of research.

### 3.1. Endometriotic Women Exhibit Altered Microbiotas

A recent study by Ata et al. sought to compare the vaginal, cervical and gut microbiota composition of women with Stage III/IV endometriosis to healthy controls [95]. Remarkably, they did indeed detect a difference at the genus level. In the cervical microbiota of endometriotic women, they found increased abundance of potentially pathogenic species including *Gardnerella*, *Streptococcus*, *Escherichia*, *Shigella* and *Ureaplasma*. Stool microbiota of the endometriotic group were *Shigella* and *Escherichia* dominant. Interestingly, they found a complete absence of *Atopobium*, a gynaecologic pathogen, in the vagina and cervix of the endometriotic group. Another study reported high incidence of *Atopobium vaginae* in women with endometrial cancer, and suggested that *Atopobium* can facilitate intracellular *Porphyromonas* infection, leading to disrupted cell regulatory functions and carcinogenic trigger [96]. Conversely, they found *A. vaginae* to have lower incidence in women with benign gynaecologic pathologies, suggesting a possible connection through a different mechanism of action, since endometriosis is also a benign gynaecologic pathology [96]. Several other studies have also found that uterine microbiota composition is altered in women with uterine diseases, including endometriosis, demonstrating its clinical relevance [97,98,99]. For example, researchers found an elevated abundance of Streptococcaceae, Moraxellaceae, Staphylococcaceae and Enterobacteriacea, and lowered Lactobacillacae in endometriotic women [97]. Recently, Hernandes et al. found that, compared with eutopic endometrium, ectopic lesions have higher microbial diversity [98]. In Wei et al.’s attempt to characterize microbiota composition and distribution along the FRT in endometriotic women, they found in conformance that the lower FRT was *Lactobacillus* dominant, and significant differences in community diversity appeared and increased from the cervix up into the endometrium and PF [99]. 

In general, studies to date have consistently found increases in BV-associated bacteria and opportunistic pathogens, and a decrease in *Lactobacillus* in the reproductive tract of endometriotic women [82,83,95].

### 3.2. Endometriosis Induces Gut Microbiota Alterations

**Figure 3:**
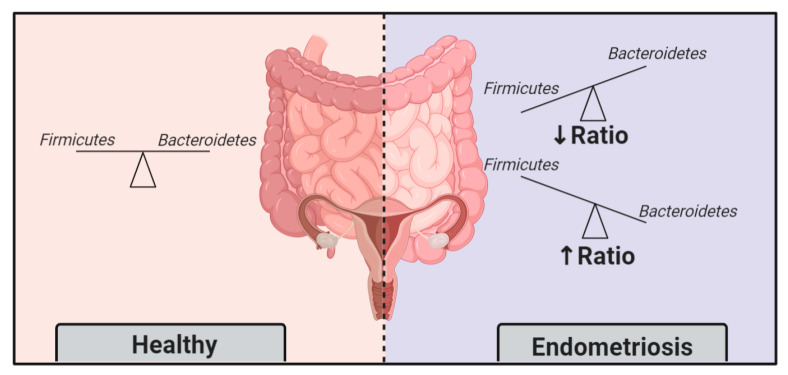
The Firmicutes/Bacteroidetes ratio, an indicator of dysbiosis, is altered in endometriosis patients.

In a study where mice were injected with intraperitoneal endometrial tissue to induce endometriosis, it was demonstrated that after 42 days of endometriotic lesion persistence, a distinct gut microbiota develops [100]. In other words, endometriosis progression was able to change the gut microbiota. Among the observed differences, the nearly doubled Firmicutes/Bacteroidetes ratio in endometriotic mice was discriminative and concrete [100]. A previous study in 2002 also found similar differences in microbiota profiles in rhesus monkeys [101]. Compared to healthy controls, monkeys with endometriosis had lower Lactobacilli and higher gram-negative bacteria [101]. The ratio Firmicutes/Bacteroidetes is widely accepted as a feature of dysbiosis (Figure 3); hence, these momentous findings support that endometriosis induces gut microbiota alterations. 

### 3.3. Faecal Microbiota Transfer Induces Endometriosis

**Figure 4:**
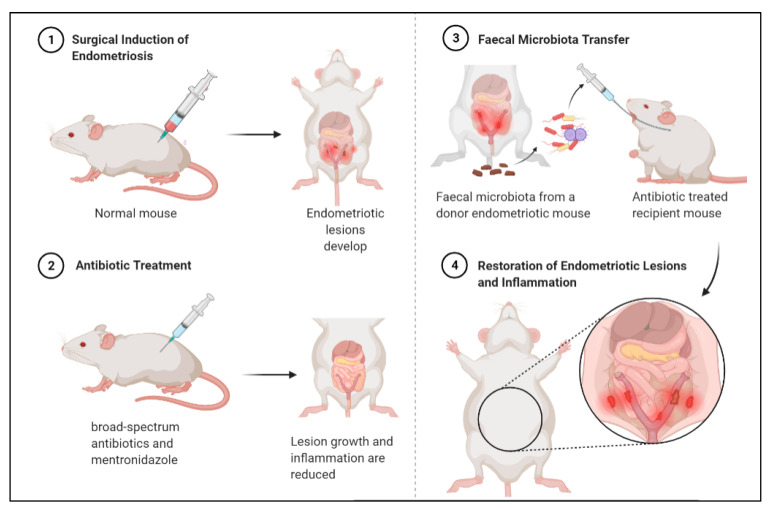
Antibiotic treatment can reduce endometriotic lesion growth and peritoneal inflammation, and subsequent faecal microbiota transfer from diseased mouse can restore lesion growth and inflammation. Mouse experiment demonstrates bidirectional relationship between endometriosis and gut microbiota.

Findings from a compelling mouse model study (Figure 4) support that a distinct gut microbiota promotes endometriosis [102]. In this study, mice were subjected to surgical induction of endometriosis, and then treated with antibiotics which reduced lesion size. Subsequently, they received faecal microbiota transfers from endometriotic mice, which restored lesion growth and associated inflammation [102]. 

### 3.4. Diet-Induced Gut Microbiota Changes Reduce Endometriosis Risk

Another interesting finding is that women with a high omega-3 polyunsaturated fatty acids (PUFAs) intake have lower risk for endometriosis [103,104]. A similar diet showed anti-inflammatory effects and suppressed endometriotic lesion formation in murine models [105,106]. It is reasonable to speculate that this can be at least partially attributed to diet-induced modification of the gut flora. Research has shown that diets high in PUFAs and probiotic supplements may alter the gut flora, and may contribute to the prevention and treatment of various diseases, including osteoporosis and obesity [107,108].

## 4. Postulated Mechanisms of Microbiota Involvement in Endometriosis

**Figure 5:**
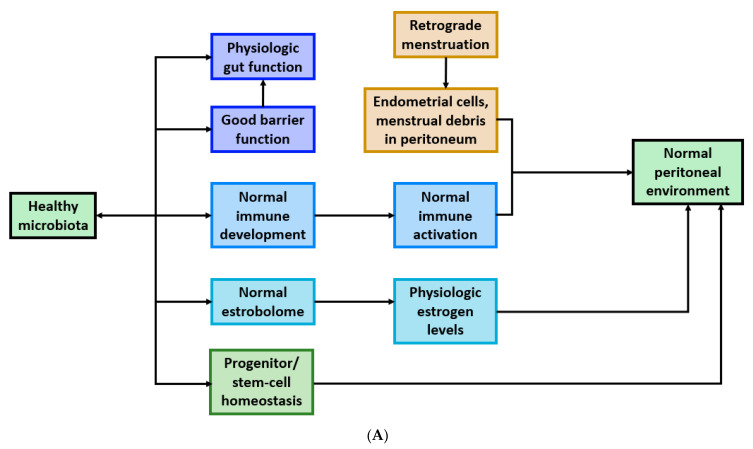

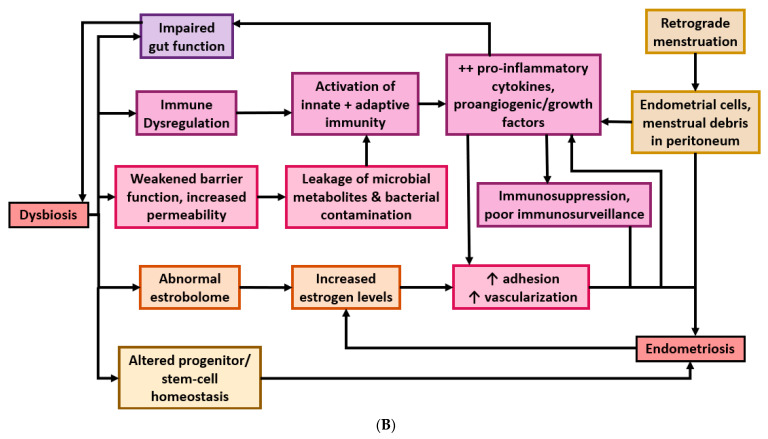
(**A**). The microbiota regulates factors involved in maintaining normal peritoneal environment and ectopic cell clearance. (**B**). Dysbiosis contributes to the dysregulation of factors driving endometriosis onset and progression.

Endometriosis is a multifactorial disease; it is impacted by activation of innate and adaptive immunity, cytokine secretion, estrogen signaling and stem and progenitor cell homeosis. Although it remains unclear whether dysbiosis causes endometriosis or vice versa, it is apparent that these connections are promising in the pursuit of diagnostic tools and therapeutics. There are several postulated mechanisms of how each of the above factors influencing endometriosis is intertwined with the microbiota, shown in Figure 5.

### 4.1. Bacterial Contamination Theory and Immune Activation

The Bacterial Contamination Theory proposes that bacterial presence in the uterine environment triggers the altered inflammatory reaction observed in endometriosis, by supplying lipopolysaccharides (LPS) which are refluxed into the PF and bind to the abundant pattern recognition receptors (PRRs) [109]. In support of this theory, Khan et al. found higher levels of *Escherichia coli* in menstrual blood of women with endometriosis, which suggests that elevated endotoxin levels in peritoneal fluid may promote TLR-4 mediated endometriosis progression [110].

Menstrual debris and endometrial fragments that arrive in the peritoneum via retrograde menstruation also release damage-associated molecular pattern (DAMP) molecules, iron and ROS [16,21,47]. These molecules lead to the activation of innate immune cells such as macrophages, neutrophils and mast cells [21,47], initiating the release of proinflammatory cytokines and angiogenic growth factors in the PF. This recruits more immune cells, promoting the inflammation and vascularisation of endometriotic lesions. The secreted interleukins also affect adaptive immune cell differentiation, thus increasing the number of T_H_17 cells which stimulate hypervascularisation [17,21]. 

While the Theory of Retrograde Menstruation may explain the arrival of endometrial tissue in the peritoneum, it does not provide an adequate explanation for why these fragments develop into endometriosis in some women and not others. At least part of this explanation may lie in the immune dysregulation of endometriotic women, who present differences in the intensity and extent of this initial immune response and in their peritoneal immune environment [1,17,21,26]. But why do some women have a far stronger immune reaction than others? It has been shown that the microbiota is a major regulator of such processes. For example, bacteria presence in the gut pre-stimulates neutrophils, pre-conditioning them for recruitment to sites of the inflammation in the peritoneum [42]. Furthermore, dysbiosis in the gut compromises barrier function, causing increased permeability and microbial metabolite leakage, which all trigger inflammatory changes [17,21]. As a result, peritoneum macrophage numbers increase and they display dysregulated function (altered phenotypes); their capacity to phagocytose newly implanted endometriotic lesions is hindered, promoting survival of lesions [17,100,111]. The presence of filamentous bacteria in the gut also induces the activation of CD4+ T cells, namely T_H_17 cells [112]. 

It is likely that the microbiota, particularly in a state of dysbiosis, may contribute to the immune activation that strengthens and prolongs peritoneal inflammation, and possibly endometriosis progression. 

### 4.2. Cytokines Affect Gut Function 

Another possible mechanism explaining how endometriosis may be inducing gut dysbiosis was elucidated when researchers discovered that endometriotic rhesus monkeys also had higher prevalence of intestinal inflammation, in addition to their gut dysbiosis [101]. The intestinal inflammation was characterised by recruitment of macrophages and monocytes, and secretion of pro-inflammatory cytokines. By now, it is well-established that peritoneal inflammation, attributable to the high local cytokine concentration, is a hallmark of endometriosis [113,114,115]. Cytokines can travel and are known to exert effects on the gastrointestinal tract, playing a role in the suppression of gastric acid secretion and gut motility [101,116,117]. This makes the internal environment less conducive to Lactobacilli species, and allows for overgrowth of gram-negative ones [101]. Although preliminary, this hypothesis offers the possible explanation that endometriosis induces gut dysbiosis by impairing gut function through cytokine activity.

### 4.3. Microbiota Composition and Estrogen Availability

Another way that the microbiota may be related to endometriosis is through the regulation of estrogen. It is known that the gut microbiota is involved in estrogen metabolism, and it was demonstrated that gut microbial richness regulates systemic estrogen levels through the action of β-glucuronidase [58,59,118,119]. β-glucuronidase converts estrogens into their active forms so that they can bind ERs (Figure 2). When there is gut dysbiosis, microbial β-glucuronidase secretion can be upregulated, which increases estrogen abundance [59]. Active estrogen in the gut can then be transported to distal mucosal sites, such as the endometrium, through the bloodstream [59]. In this way, the gut microbiota regulates estrogen homeostasis in both intestinal and distal sites. It is suspected that gut microbiotas of endometriosis patients have a larger number of β-glucuronidase producing bacteria [59]. In fact, the implicated Firmicutes/Bacteroidetes ratio observed in endometriotic women may be acting through the dysregulation of estrogen metabolism, since microbes in these phyla possess glucuronidase-related genes [120,121]. 

Gut dysbiosis can also contribute to changes in the metabolome, which may manifest in increased levels of neuro-active metabolites such as serotonin, glutamate, short chain fatty acids (SCFAs) and gamma-aminobutyric acid [61,63,64,122,123,124]. These metabolites travel to the brain and stimulate neural receptors, including GnRH neurons, ultimately upregulating ovarian secretion of estrogen through sequential hormone signaling [61,62,65].

Conversely, estrogen levels have also been shown to impact the microbiota [97]. A study found that suppression of estrogen with GnRH-agonist modified uterine microbiota composition [97], while increased estrogen promoted *Lactobacillus*-dominance in the genital microbiota [125]. The altered estrogen availability and signaling in endometriosis, resulting from imbalances in the estrobolome, upregulation of estrogen-synthesis enzymes and abnormal estrogen receptor expression, could therefore have implications on the genital microbiota composition [97]. This could lead to loss of healthy microbiota, and increase risk for conditions such as BV, which are a known gateway to more severe gynaecologic diseases, such as endometriosis, by increasing inflammatory cytokine concentration and damaging epithelial barriers [59,74,83,84,85,87,88,89].

### 4.4. Microbiota Regulates Progenitor and Stem-Cell Homeostasis

Research on the involvement of stem cells and progenitor cells derived from the bone marrow in endometriosis is accumulating. Human endometrial tissue is highly dynamic and undergoes regular cyclic regeneration, and therefore contains a repository of progenitor stem cells [126]. In patients with endometriosis, normal stem cell mobility and trafficking to the uterus is altered [126]. They migrate to ectopic sites via the bloodstream, allowing for uncontrolled growth of endometrial tissue beyond the normal uterine environment, and contributing to endometriosis [126]. Interestingly, the gut microbiota composition has been reported to correlate with stem cell proportions in the bone marrow, suggesting it may be involved in modulating stem-cell homeostasis [127], and thus the pathophysiology of endometriosis.

The present explanations still require extensive research; many are speculations arising from interesting observations, but there is a paucity of robust studies to demonstrate causal relationships.

## 5. What Can This Mean for Endometriosis Care

It is evident that bacterial presence in both the gut and uterus plays a major role in endometriosis. But what does this mean for patients? Could the modulation of the microbiota be a therapeutic or preventative approach? Could certain microbial compositions or the presence of microbiota-based biomarkers be used as screening or diagnostic tools?

### 5.1. Gynaecologic and Obstetric Applications of Microbiota Modulation

Microbiota modulation through antibiotics is already broadly applied in the field of gynaecology and obstetrics [128]. In accordance with ample research showing uterine dysbiosis threatens fertility and pregnancy outcomes, it is suggested that intervention options such as uterine lavage or antibiotics to eradicate microbes or pro/prebiotics and improve the microbiota could be valuable [128]. In a recent clinical setting, broad-spectrum antibiotics and pre/probiotics were employed to achieve *Lactobacillus*-dominant uterine microbiota (from previously non-*Lactobacillus*-dominant), which led to higher pregnancy rates [129] — A hopeful finding that encourages further investigation of this approach. Moreover, many studies show that treatment of chronic endometritis with antibiotics leads to improved reproductive outcomes [130,131,132,133]. 

### 5.2. Treating Endometriosis with Antibiotics

Antibiotics may be a promising approach for treating endometriosis. In fact, broad-spectrum antibiotic treatments have demonstrated efficacy for treating endometriosis in animal models [102]. A recent study found that use of broad-spectrum antibiotics inhibited ectopic lesions, while treatment with metronidazole significantly decreased inflammation and reduced lesion size, possibly by lessening Bacteroidetes presence [102]. Peculiarly, treatment with neomycin did not produce the same results, indicating that lesion-growth-promoting bacteria are metronidazole-sensitive and neomycin-resistant [102].

### 5.3. Treating Endometriosis with Probiotics 

Alternatively, probiotic intervention, the administration of live microorganisms, could be another effective approach [128]. For instance, in randomised, placebo-controlled trials, oral administration of *Lactobacillus* has been shown to ameliorate endometriosis-associated pain in women [134,135], and reduce endometriotic lesions in mice by increasing IL-12 concentration and NK cell activity [128,136,137]. Dysbiosis and endometriotic-inflammation leads to impaired NK cell activity, and the probiotic treatment reversed this immune dysregulation. *Lactobacillus* probiotic treatment not only improved endometriosis, but is also capable of preventing its growth in rats [136]. These impressive frontiers warrant research and testing. 

### 5.4. A Mechanism for Known Treatments 

New research is showing that some known endometriosis treatments may have actually been working through gut microbiota modulation [138]. For example, letrozole, an aromatase inhibitor that reduces estrogen levels and Shaofu Zhuyu decoction (SFZYD), a traditional Chinese medicine that inhibits cellular proliferation, promotes apoptosis, and reduces angiogenesis in ectopic endometrial tissues, have been shown to inhibit the progression of endometriosis and reduce inflammation in mice [138]. In a 2020 study, it was found that both letrozole and SFZYD exert their therapeutic effect in part through restoration of the gut microbiota; they both attenuated the Firmicutes/Bacteroidetes ratio, which was elevated in the untreated endometriotic group, and restored α-diversity and Ruminococcaceae abundance in the gut microbiota [138]. Loss of Ruminococcaceae may exacerbate peritoneal inflammation, as it may be negatively correlated to apoptosis of intestinal epithelial cells and murine IL-6 levels [139]. Therefore, these treatments at least partially function through restoring gut microbiota health. 

### 5.5. Side Effects and Challenges 

Unfortunately, the use of antibiotics is known to introduce off-target effects and new interventions such as pre/probiotics have poorly understood pharmacological mechanisms with difficult-to-prove clinical efficacy. For example, it is well known that routine or overuse of antibiotics increases the risk of antimicrobial resistance, which is currently one of the largest threats to global human health [140]. Furthermore, the core FRT microbiota should be well characterised before applying any such treatments [128]. Antibiotics are widely effective in treating infection by reducing abundance or eliminating pathogenic species, however its use alters microbial community profiles, and can create lasting disruptions to healthy microbiotas [141,142]. A study examining the urinary microbiotas of women during BV and after oral administration of metronidazole found that while it was an effective treatment, it also significantly decreased the Shannon diversity, an effect which persisted for up to 28 days [141]. Furthermore, the antibiotic treatment created complex changes to the microbiota composition, and healthy microbial community could not be restored [141]. Compared to BV, dysbiosis related to endometriosis is far less studied, and therefore the convoluted effects of antibiotic treatments are largely undetermined. The use of antibiotics to treat endometriosis still requires extensive research, but is an area of potential. There are many unanswered questions regarding its practical use, and the diagnosis of an “abnormal uterine microbiota” to indicate such treatment remains a major hurdle to overcome. 

### 5.6. Opportunities for Diagnostics 

Another appealing application of the microbiota-endometriosis relationship lies in diagnostics. So much is yet to be learned about the mechanisms involved, but based on the current state of knowledge that there is an appreciable difference in the microbiotas of women with endometriosis, we can imagine its potential value as a diagnostic or screening tool. Significant differences in the community diversity of cervical microbiota in endometriotic women indicate that cervical samples may be used as an endometriosis risk indicator [99]. A recent study found for the first time that vaginal microbiome profiles could be successfully used to predict the rASRM (revised American Society for Reproductive Medicine) stage of endometriosis [143]. These exciting findings hopefully incite further research into non-invasive diagnostics and screening tools, as traditional techniques are limited and remain a challenge today.

## 6. Limitations and Future Directions 

**Figure 6:**
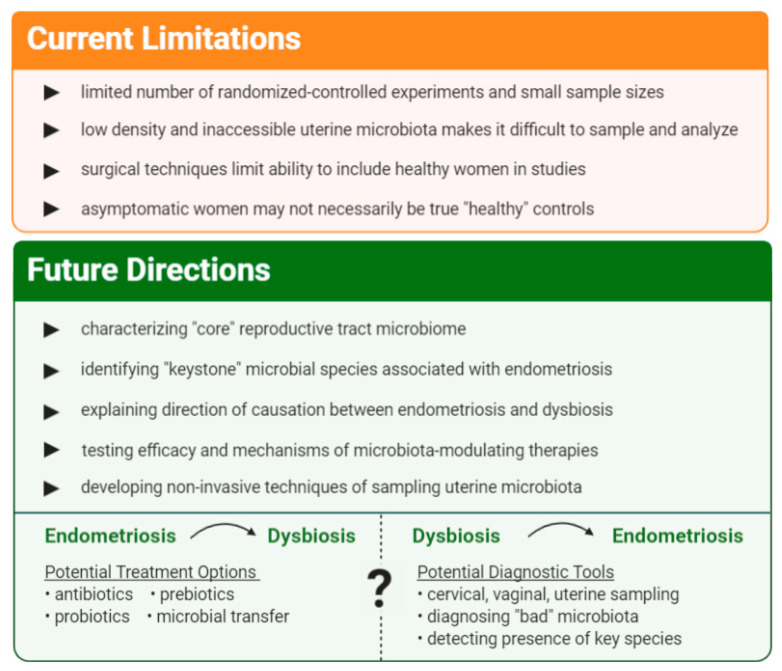
Summary of current limitations, future directions and possible outcomes.

As a newly emerging area of research, the relationship between and the impact of the microbiota on endometriosis is still largely enigmatic. Within the current pool of literature, many studies were performed on small sample sizes with a lack of robust randomised-controlled experiments. Different techniques of analyzing microbial composition and varying levels of resolution may produce disparate results, contributing to the already challenging feat of achieving a “core” microbiome consensus. Furthermore, current standards of diagnosing endometriosis and sampling the uterine microbiota involve invasive procedures; as a result, ethical inclusion of asymptomatic healthy populations in studies is limited. Nonetheless, the vast implications of this research on prevention, diagnosis and treatment of endometriosis hold promise (Figure 6). 

Future investigations should include not only the profiling of a core FRT microbiome, but the identification of “keystone” microbial species associated with endometriosis and their mechanisms of exerting influence, such as through disruption of healthy FRT microbiota, activation of immune responses or secretion of microbial metabolites [27,74]. Research should also aim to understand the causational direction between dysbiosis, estrogen metabolism and endometriosis, as well as site-specific host-microbiota interactions, possibly elucidating how it applies to other gynaecologic or estrogen-mediated diseases [59,74]. Certainly, efforts to test the efficacy and unravel the mechanisms of different microbiota-modulating therapies, as well as non-invasive FRT microbiome sampling techniques, should be made [17,128].

## 7. Conclusions

Dysbiosis in the gut and female reproductive tract disrupts normal immune function, leading to inflammatory responses by elevating proinflammatory cytokines, compromising immunosurveillance and altering immune cell profiles. This immune dysregulation can progress into a chronic state of inflammation, creating an ideal environment conducive to increased adhesion and angiogenesis, which may drive the vicious cycle of endometriosis onset and progression. Recent studies have demonstrated both the ability of endometriosis to induce microbiota changes, and the ability of antibiotics to treat endometriosis. In general, endometriotic microbiotas are associated with diminished *Lactobacillus* dominance and the elevated abundance of potentially pathogenic species. The Bacterial Contamination Theory and immune activation, cytokine-impaired gut function, aberrant estrogen metabolism and signaling, as well as aberrant progenitor and stem-cell homeostasis, are possible explanations for how dysbiosis is implicated in this disease. Although preliminary, antibiotic and probiotic treatments have demonstrated efficacy in treating endometriosis, and FRT microbiota sampling has successfully predicted disease risk and stage. Extensive research is still required, particularly to characterise the “core” microbiota and elucidate mechanisms behind the microbiota-endometriosis relationship.

## Author Contributions

Conceptualization, I.J. and M.A.B.; Investigation, I.J.; Writing —original draft preparation, I.J.; Writing —review and editing, I.J., M.A.B.; Visualization, I.J.; Supervision, M.A.B., P.J.Y., C.A.; Project administration, I.J., M.A.B. All authors have read and agreed to the published version of the manuscript.

## Figures and Tables

**Table 1:** Dysregulation of peritoneal innate and adaptive immunity creates environment conducive to endometriosis onset and progression.

	Macrophages	Neutrophils	NK Cells	T Cells	B Cells
	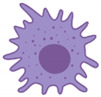	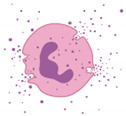	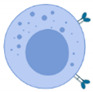	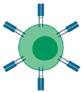	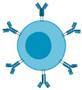
**Immune Dysregulation**	High numbers in PF and endometriotic lesions ↓ phagocytosis ↑ cytokine secretion (TNF-α, IL-6, IL-1β, NF-κB, VEGF) Altered phenotype: more proinflammatory	Elevated numbers in PF Preconditioned by bacterial presence Recruited to lesions by IL-8	Impaired by aberrant immune environment (IL-6, TGF-β) Altered activating/ inhibitory receptor pattern	Altered subset proportions ↑ T_H_2 ↑ T_H_17 Increased IL-17 secretion	Produce anti-endometrial autoantibodies ↑ IL-6 ↑ IL-17
**Implications on Endometriosis**	↓ ability to eliminate refluxed endometrial tissue Promote further immune activation and inflammation Lesion growth & vascularization	Involved in early lesion formation Promote angiogenesis	Reduced cytotoxic capacity = ↓ immunosurveillance Lesion survival	Suppressed cell-mediated immunity = ↓ immunosurveillance Stimulate production of proinflammatory cytokines and pro-angiogenic factors Endometriosis progression	Contribute to inflammation, disease progression Role is unclear

## Data Availability

Not applicable.
